# Care considerations in medical assistance in dying for persons with mental illness as the sole underlying medical condition: a qualitative study of patient and family perspectives

**DOI:** 10.1186/s12888-024-05541-5

**Published:** 2024-02-14

**Authors:** Vicky Stergiopoulos, Hamer Bastidas-Bilbao, Mona Gupta, Daniel Z. Buchman, Donna E. Stewart, Tarek Rajji, Alexander I. F. Simpson, Mary Rose van Kesteren, Vivien Cappe, David Castle, Roslyn Shields, Lisa D. Hawke

**Affiliations:** 1 Centre for Addiction and Mental Health, Toronto, Ontario Canada; 2 University of Toronto, Toronto, Ontario Canada; 3 Centre hospitalier de l’Université de Montréal, Montréal, Québec Canada; 4 University Health Network, Toronto, Ontario Canada; 5 University of Tasmania, Hobart, Tasmania Australia; 6 Statewide Mental Health Service, Hobart, Tasmania Australia

**Keywords:** Medical assistance in dying, Mental illness, Lived experience, Patient and family perspectives, Canada

## Abstract

**Background:**

Persons with mental illness as their sole underlying medical condition are eligible to access medical assistance in dying (MAiD) in a small number of countries, including Belgium, the Netherlands, Luxemburg and Switzerland. In Canada, it is anticipated that people experiencing mental illness as their sole underlying medical condition (MI-SUMC) will be eligible to request MAiD as of March 17th 2024. To date, few studies have addressed patient and family perspectives on MAiD MI-SUMC care processes. This study aimed to address this gap and qualitatively explore the perspectives of persons with lived experience of mental illness and family members on care considerations during MI-SUMC implementation.

**Methods:**

Thirty adults with lived experience of mental illness and 25 adult family members residing in Ontario participated in this study. To facilitate participant engagement, the semi-structured interview used a persona-scenario exercise to discuss perspectives on MAiD MI-SUMC acceptability and care considerations. Framework analysis was used to inductively analyze data using NVivo 12 Pro. Steps, processes, or other care considerations suggested by the participants were charted in a framework matrix after familiarization with the narratives. Key themes were further identified. A lived-experience advisory group participated in every aspect of this study.

**Results:**

Six themes were developed from the patient and family narratives: *(1) Raising MAiD MI-SUMC awareness; (2) Sensitive Introduction of MAiD MI-SUMC in goals of care discussions; (3) Asking for MAiD MI-SUMC: a person-focused response; (4) A comprehensive circle of MAiD MI-SUMC care; (5) A holistic, person-centered assessment process; and (6) Need for support in the aftermath of the decision.* These themes highlighted a congruence of views between patient and family members and described key desired process ingredients, including a person-centred non-judgmental stance by care providers, inter-professional holistic care, shared decision making, and the primacy of patient autonomy in healthcare decision making.

**Conclusions:**

Family and patient perspectives on the implementation of MAiD MI-SUMC offer important considerations for service planning that could complement existing and emerging professional practice standards. These stakeholders’ perspectives will continue to be essential in MAiD MI-SUMC implementation efforts, to better address the needs of diverse communities and inform improvement efforts.

**Supplementary Information:**

The online version contains supplementary material available at 10.1186/s12888-024-05541-5.

## Background

Most jurisdictions where medical assistance in dying (MAiD) is legalized have adopted end of life legal frameworks, and do not contemplate eligibility for chronic health conditions such as mental disorders, where death is not reasonable foreseeable. Persons with mental illness as their sole underlying medical condition are currently eligible to access MAiD in a small number of countries whose laws consider irremediable chronic health conditions, including Belgium, the Netherlands, Luxemburg and Switzerland. The reported number of such MAiD deaths in these countries remains small, but has been increasing over time [1, 2]. It is estimated that in 2021, 0.9% and 1.5% of all MAiD deaths in Belgium and the Netherlands respectively were of people with a mental illness as their sole underlying medical condition [3, 4].

The ethical permissibility of MAiD where a mental illness is the sole underlying medical condition (MAiD MI-SUMC) remains a subject of much debate in the international literature and among professional bodies [1, 5–13]. Those in support of MAiD for mental illness argue that mental disorders should not be treated differently to other health conditions in countries where medically assisted deaths for chronic health conditions are legalized, and highlight the primacy of patient autonomy [5, 13, 14]. Others, irrespective of whether they are for or against MAiD, underscore the difficulty in establishing irremediability, in differentiating a MAiD request from suicidality, and the impact of limited access to care and the broader social determinants of health on people with mental disorders [5, 9, 15, 16].

In Canada, it is anticipated that people experiencing a mental illness as their sole underlying medical condition will be eligible to request MAiD as of March 17th, 2024 [17]. Under the current legal framework introduced by Bill C-14 [18], and updated by Bill C-7 [19], MAiD requests can be made by adults eligible for health insurance in Canada who experience a ‘grievous and irremediable’ medical condition and who have capacity to make treatment decisions. Informed consent for MAiD is required after applicants are informed of other available treatments as well as immediately prior to the delivery of MAiD [18, 20]. Safeguards include submitting a witnessed written request, at least two independent professional opinions, and the right to withdraw the request at any time. For those whose death is not reasonably foreseeable, additional legislated safeguards include a mandatory minimum of 90 days between the onset of the assessment and MAiD provision, a consultation with a physician or nurse practitioner with expertise in the person’s condition if the two assigned assessors lack such expertise, and agreement that all reasonable and available means to relieve suffering have been discussed and seriously considered [19, 20] (Table S1). At this time, it is not known whether additional safeguards for MAiD MI-SUMC will be put in place in Canada [21]. Some clinicians from jurisdictions where MAiD MI-SUMC is available have argued for a stricter practice framework for those with mental disorders, with continued recovery-oriented care in parallel with MAiD MI-SUMC evaluation by at least two psychiatrists, and a prospective multi-expert panel to review and manage MAiD requests [22, 23].

While MAiD practice standards are available and training resources for health providers are in development in Canada, the latter with input from patients, complementing these resources with the perspectives of patients with mental illness and family members is central to delivering high quality programs and practices that meet their needs and preferences. The scant available literature on patient and family perspectives on MAiD delivery to date highlights care coordination and patient-centered approaches as central to high-quality care [24]. Recent studies have also highlighted the need for holistic, interdisciplinary care, relationship building and anticipatory guidance throughout the process, as well as bereavement supports for family and loved ones, and dedicated space for MAiD provision [25–27]. Research on patient and family perspectives on how to implement person-centered MAiD MI-SUMC programs is sorely lacking. This study used qualitative methods to explore the views of persons with mental illness and family members on MAiD MI-SUMC implementation and care processes. Persons with lived experience of mental illness and family members were engaged in every aspect of the study, including design, data collection and analysis, and manuscript preparation. Findings, grounded in the needs and preferences of these primary stakeholder groups, can inform the development and ongoing improvement of MAiD MI-SUMC programs, complementing professional perspectives and experiences.

## Methods

Leveraging the Canadian Institutes of Health Research Strategy for Patient-Oriented Research (SPOR) [28] we engaged a study advisory group inclusive of two adults with lived experience of mental illness and two family members of persons with lived experience of mental illness. This lived-experience advisory group contributed to every aspect of the study, from planning to recruitment, analysis and reporting, with two of the advisors co-authoring this manuscript. In acknowledgement of the value of the lived experience as a source of knowledge, we adopted a critical realist ontology [29, 30] and contextualism [31] as our epistemology. We wanted to recognize that the experiences and understandings of the participants are not uniform nor isolated from their sociocultural context; researchers and knowledge construction practices are also influenced by the sociocultural contexts in which they are situated. Saturation of data [32] was not pursued, as our ontological and epistemological positions focused on exploring realities and forms of knowledge that cannot be presumed static or to fall within strictly defined boundaries. The results are reported in accordance with the Standards for Reporting Qualitative Research (SRQR) [33]. Throughout the research process we were each mindful of our positionality, background and experiences, which included diverse social locations in regards to power, privilege and access to resources. Our team included scientists, clinicians, including MAiD assessors, people with lived experience, and trainees.

We recruited 30 adults who self-identified as having a mental illness and 25 adults who self-identified as family members. Eligibility criteria included age over 18, residency in Ontario, and having experienced mental illness or being a family member of an individual with mental illness. For this study, we used a broad definition of family, going beyond the family of origin to include individuals closely connected to a person with mental illness. Participants were recruited across Ontario between April and December 2022. Recruitment targets aimed to balance pragmatic considerations with the opportunity to enhance diversity of perspectives and experiences and promote information redundancy. Recruitment strategies included e-flyers, circulated among mental health community organizations across Ontario, study ads on Facebook, use of the research registry of the Centre of Addiction and Mental Health in Toronto, Ontario (inclusive of potential participants who consented to be contacted about research opportunities), and contacts of our lived-experience advisory group. Following an assessment of eligibility, grounded in discussions of the underlying mental health problems and their impact, and completion of an informed consent discussion, participants provided e-consent and answered a brief demographic questionnaire in interview format. Documentation was managed via Research Electronic Data Capture (REDCap) [34]. Qualitative interviews of approximately one hour took place via phone or video call using Cisco Webex. A post-doctoral research fellow [HB] managed all study procedures, including participant recruitment, data collection and analysis. The study received approval from the Research Ethics Board of the Centre for Addiction and Mental Health in Ontario, Canada.

The semi-structured interview guide [35], based on a persona-scenario exercise (PSE) [36] to facilitate participant engagement, was developed using participatory approaches, including input from our lived-experience advisors. In PSE [36], participants are invited to construct a persona and supplement details based on interview prompts. This flexible approach guides participants to discuss the difficult topic in a way that is externalized and less triggering rather than personally referent. After introducing study participants to the legal framework guiding MAiD in Canada that was current at the time of the study, the interview guide first explored participant’s perspectives on MAiD MI-SUMC. The interviewer then asked participants to imagine a persona living with mental illness who, in their view, might be inclined to request MAiD MI-SUMC. Participants were subsequently invited to comment on the process of assessing eligibility for MAiD MI-SUMC and scenarios where it might be acceptable or unacceptable for the constructed persona to access MAiD MI-SUMC. Finally, the interview explored participants’ perspectives on suicide and suicidality in MAiD MI-SUMC. The interview guide was piloted and a member of our lived-experience advisory group provided feedback on the pilot interview video recording before finalizing the interview guide. Interviews were audio recorded, transcribed and proofed for accuracy. Participants received CAD $30.00 in a physical or digital gift card for their participation.

Transcript sections describing the process of accessing MAiD MI-SUMC and assessing MAiD MI-SUMC eligibility were analyzed using Framework Analysis [37, 38], a methodology frequently used in health settings to systematically organize large data sets by mapping the data onto a matrix that can be used to create summaries from individual cases or summaries derived from codes or topics across the entire dataset. As we were interested in exploring relatively concrete information [29] such as steps, procedures, or actions—that participants found important to be included in a potential implementation of MAiD MI-SUMC—the framework analysis seemed to be the most appropriate approach. The analysis included an initial stage of familiarization with the data; an iterative process of coding, developing the analytic framework, and applying it to subsequent transcripts; summarizing of cases and topics; and development of key themes and interpretations. Data analysis was led by HB, and was supported by a data analysis working group that met biweekly to discuss emerging codes and themes, and resonance with practice and lived experience. NVivo 12 Pro was used for data management, coding and charting onto a framework matrix. Microsoft Excel 2016 was used to track changes made to the codes and analytic framework throughout the study. Shared perspectives and divergent views within individuals and across groups were examined in the last phase of the analysis, informing the grouping of themes into higher level categories and articulating linkages between them.

## Results

Participant demographic characteristics are included in Table 1.

**Table 1:** Participant characteristics

Characteristic		Patients sample (N = 30) n (%)	Family member sample (N = 25) n (%)
Age	18–34	12 (40.0)	7 (28.0)
	35–54	10 (33.3)	8 (32.0)
	55+	8 (26.7)	10 (40.0)
Gender	Man	12 (40.0)	3 (12.0)
	Woman	15 (50.0)	20 (80.0)
	Transgender/Gender non-binary	3 (10.0)	2 (8.0)
Ethnicity	White	18 (60.0)	18 (72.0)
	Racialized^1^	12 (40.0)	7 (28.0)
Born in Canada		22 (73.3)	21 (84.0)
Identifies with religious or spiritual beliefs		16 (53.3)	16 (64.0)
Housing status^2^	Stable housing	21 (70.0)	n/a
	Precarious housing	9 (30.0)	n/a
Physical health	Good to excellent	19 (63.3)	19 (76.0)
	Fair to poor	11 (36.7)	6 (24.0)
Mental health	Good to excellent	16 (53.3)	18 (72.0)
	Fair to poor	14 (46.7)	7 (28.0)
Mental illness^2^	Trauma experiences or trauma- and stressor-related disorders	11 (36.7)	n/a
	Anxiety disorders or related symptoms	15 (50.0)	n/a
	Obsessive-compulsive and related disorders, or related symptoms	9 (30.0)	n/a
	Depressive disorders or related symptoms	19 (63.3)	n/a
	Bipolar and related disorders, or related symptoms	5 (16.7)	n/a
	Personality disorders	6 (20.0)	n/a
	Schizophrenia spectrum and psychotic disorders, or related symptoms	6 (20.0)	n/a
	Neurodevelopmental disorders, or related symptoms	5 (16.7)	n/a
	Other disorders or symptoms^5^	4 (13.3)	n/a
Substance use^3^	Past or present use of alcohol, including problematic use	6 (20.0)	n/a
	Past or present use of other substances, including problematic use, or substance not specified^2^	4 (13.3)	n/a
History of self-harm or suicidality^3^		20 (66.7)	n/a

^1^Reported in aggregate due to small cell sizes

^2^Not all participants described mental illness using specific diagnostic labels. This information is therefore grouped into categories of mental disorders and related symptoms. Percentages exceed 100% due to reported comorbidities

We generated six themes from the patient and family narratives: *(1) Raising MAiD MI-SUMC awareness - Public information and patient psychoeducation; (2) Sensitive introduction of MAiD MI-SUMC in goals of care discussions; (3) Asking for MAiD MI-SUMC: a person-focused response; (4) A comprehensive circle of MAiD MI-SUMC care; (5) A holistic, person-centered assessment process; and (6) Need for support in the aftermath of the decision.* We present participant perspectives under each theme and subtheme, and highlight areas of divergence, where they emerge. Representative quotes are followed by a number preceded by P or F for person with mental illness or family participant, respectively. Numbers were assigned sequentially and are not related to study records.

### Raising awareness– public information and patient psychoeducation

Patient and family perspectives fully aligned in highlighting the need for public education around the legal framework, the MAiD MI-SUMC request and assessment process, and safeguards in place for individuals where mental illness is the sole underlying medical condition.

Both patient and family member participants spoke to the need for raising public awareness and making broadly available patient-facing information, including pamphlets in providers’ offices. Furthermore, they commented that clinician-led psychoeducation for patients around MAiD MI-SUMC should outline eligibility criteria, steps for submitting a request, details on the assessment process, anticipated timelines, and the different options for administering MAiD. As this participant commented:

*“So, if [the imaginary person] requests information about MAID, then I think the service provider should be able to answer any and all questions [the imaginary person] has about the program itself, what it entails, what the criteria is, if there’s a waitlist, the risks, you know, versus benefits of this. If there would be any costs, maybe not with the program itself, because we have great healthcare, but you know, is there anything else in preparation for– or something? Is there anything that people have to take in consideration to prepare?” (F1)*.

They also described the need for education about other forms of relief, including new and emerging or experimental treatments, as this participant put forward:

*“Give [the imaginary person] the knowledge that treatments could be developed in the future that could help his condition. So, if [the imaginary person] goes the route of MAiD, he won’t be able to access any of those new treatments if they became available.” (P1)*.

Finally, they highlighted that MAiD MI-SUMC should not be described “as a quick fix” (F1), but rather providers should discuss the pros and cons, including its irreversibility once administered, and MAiD’s impact on others. As this participant commented:

*“That it’s something that you should consider only after you’ve tried a bunch of different other things [that] haven’t helped or they’ve made it worse– that there might be people whose lives are going to be impacted by [the imaginary person’s] decisions.” (P2)*.

### Sensitive introduction of MAiD MI-SUMC in goals of care discussions

Opinions among both patient and family member participants were divided on the acceptability of health providers introducing MAiD MI-SUMC as an option in goals of care or treatment discussions. Several participants voiced that MAiD conversations should only be initiated by patients themselves, as this participant expressed:

*“No, absolutely not. No. I think it should be something– I think they should provide the information if they’re asked about it, because I guess they legally probably have to, or refer to whatever would be the right assessment MAiD agency, whatever. But I don’t think that they should mention it as an option.” (P3)*.

These participants emphasized that providers should focus on helping make their patients’ lives livable, and raised concerns that with MAiD MI-SUMC presented as an option, patients might forego potentially effective treatments. Other participants suggested that a health care provider could mention MAiD MI-SUMC in the context of unbearable suffering, a trusting patient-provider relationship, and in a late stage of illness that has not responded to available treatments after “many years” (P4), if in alignment with patient values and goals of care. Yet other participants raised concerns about health care providers discussing MAiD as an option prematurely, as this participant described:

*“I don’t want it thrown at people, with all this foolish community uproar about it.” (P5)*.

Or, as another voiced:

*“Again, it depends on the context that somebody is having. If the clinician thinks that– like if they’re hearing from somebody that they’ve tried other treatments or they’ve been struggling for a long time, then I think that it is only fair to let people know about it. I don’t think that simply stating it would influence someone to choose it.” (P4)*.

Many participants highlighted that MAiD MI-SUMC is not a treatment, and should not be presented as a treatment option, but rather discussed as a last resort to alleviate suffering once all other options have been exhausted. As this participant voiced:

*“Death is not a treatment” (F2)*.

And another participant described:

*“I don’t think MAiD is a treatment option. I think MAiD is an end of treatment option[s]… there’s no treatment, and this is your option. You live with it or you don’t live with it; that’s the option.” (F3)*.

### Asking for MAiD: a person-focused response

Both patient and family member participants expressed that providers would need to provide both information and guidance to patients planning a formal MAiD request, within person-centred encounters that are trauma-informed, validate experiences of suffering, and convey active listening and hope for recovery. As this participant highlighted:

*“Sympathetically understanding and explaining the process of what would happen, and how it would work. […] Maybe that might be enough to make the person stop and think, ’well, maybe, maybe not today.’ And if that’s the case, then I think the person that they’re talking to about MAiD should say, ‘well, then what is it that we could help to make you say this tomorrow, and the next day and the day after that?’.” (F4)*.

Participants expressed concern that some providers may not want to offer assistance in accessing MAiD MI-SUMC due to their personal or religious views, or that they may discourage patients or stigmatize MAiD MI-SUMC. They highlighted the importance of a neutral stance, with providers not trying to persuade patients in either direction, focusing on patient needs and preferences:

*“Of course, I understand we are all human, so we all have our own biases. However, when you’re working in a profession that deals with extremely vulnerable sensitive topics, where somebody’s life is in your hands — and, you really need to put yourself aside and honour your patients’ needs the most.” (P6)*.

They also identified respectful communication in plain language, openness, empathy, genuine collaboration and shared decision making as central to positive experiences. As one participant described:

*“Yeah, the healthcare worker should speak very carefully” […] “hear-out [the imaginary person] — what his opinions are; so, listen respectfully” […] “That probably sympathized with him a lot, said she is sorry for his situation, his diagnosis, and listened respectfully and professionally, told him about what MAiD was about and answered any questions he had.” (P7)*.

Participants stressed the importance of non-judgmental interactions and trust and respect in patient perspectives and experiences. As these participants expressed:

*“First and foremost, absolutely no judgments in any sense of ‘are you sure that’s what you want?’ Or ‘you’re so young’ or ‘you have kids’, or like — none of that” (P8)*.

And:

*“And once again, not judging, but really working with [the imaginary person] to ensure that the decision she’s making is based on the facts of her illness and consequences to her life” (F5)*,

And:

*“Be open minded that [the imaginary person] might know what’s best for [themselves].” (P9)*.

Participants expressed hopes that providers would actively listen and explore patient needs and preferences. Finally, they identified the importance of providers enhancing their understanding of issues such as poverty, loneliness and marginalization, so they can better appreciate what could be addressed to alleviate suffering. As this participant related:

*“Well, I think it’s important to listen to [the imaginary person], because it’s his life and his journey and it’s hard for other people to put their values on things if [the imaginary person] does not have the support that other people have, or the finances, or the medication working, or friends, or family to help him.” (F6)*.

### A comprehensive circle of MAiD care

Patients and families were similarly aligned in discussions of the most appropriate MAiD MI-SUMC assessment team composition and of the role of families and social supports in the assessment process. Both participant groups highlighted the primacy of patient preferences in guiding the process, as this participant explained:

*“If [the imaginary person] comes in and is like, ‘I don’t want to discuss this with anyone else, I don’t want to bring in my psychiatrist, I don’t want to bring in my family, this is my own decision,’ then, the family doctor sees that [the imaginary person] is in his right mind and understands and is able to comprehend what MAiD is, then, they shouldn’t bring anybody else in. It should be based on what the client wants.” (F7)*.

Main subthemes, focused on the assessment team and the role of family and social supports, are further discussed below:

### *An Expert Interdisciplinary Assessment Team*

Many participants emphasized the need for assessors to have deep knowledge and understanding of mental health and mental illness. In this context, several participants questioned whether family physicians or nurse practitioners “have the expertise” (P10) needed or “are qualified” (P11) to assess the adequacy of past mental health treatments and what treatments could yet be tried.

Most participants expressed strong preferences that assessors be psychiatrists, supported by a team or a panel of additional providers to address the psychiatric, psychological, social, spiritual and relational aspects of patients’ experiences and suffering. As this participant opined:

*“I think it shouldn’t be down to one physician. I think it should be a multi-disciplinary approach, so for example a physician might not necessarily look into socio-demographics, whereas a social worker would be more inclined to look at that. So, I would take it from [the] perspective of, you know, physician, a social worker, a nurse, even a physical therapist, an occupational therapist, just very multi-disciplinary– just to assess [the imaginary person] and her capabilities from different positions, because medication and therapy aren’t the only ways to improve her quality of life.” (P12)*.

The importance of outside consultations was also commented upon by several participants, including consultations with the applicant’s primary mental health care providers and case workers or counselors. As these participants described:

*“Other references to see whether someone has been assessed, who they’ve been assessed by, what that person has to say.” (P3)*.

And:

*“There could be exceptions where, say, the doctor wants to consult with a clinician or like a counsellor, or somebody who has more knowledge, ‘OK, in your opinion, how are you feeling about this person? Do you want to have a conversation with them?’”(F1)*.

They further identified that those providers biased against MAiD MI-SUMC should not be involved in the assessment process, for example:

*“There are providers who have their own agendas and their own set of values of, like, how certain doctors won’t perform abortions because they’re religious. I kind of feel like there shouldn’t be a conversation with that provider.” (P8)*.

In addition to professionals, they highlighted the support that peers or advocates could offer, as this participant described:

*“And if there’s a peer involved or peers then they can speak to– they can, they would have a unique understanding to [the imaginary person’s] situation that they could say something, add something to that assessment.” (P13)*.

Finally, participants commented on the importance of assessors having knowledge of the patient directly, or by connecting to their support teams, as this participant described:

*“I think…, with consent of the patient, yes. I think that the other — like, anyone that the patient trusts– I think should be involved. So, yes, like counsellors, regular family doctor, psychiatrist, psychologist, any person that touches their life that [the imaginary person] would include. I think it should be patient-led. And yes, I think they should have to make sure they consult with all the people that the patient identifies.” (F8)*.

#### Offering an appropriate role for families and social supports, based on the patient’s wishes

The majority of both patient and family member participants underscored patient choice in involving family members in the assessment process, as these participants identified:

*“So, I think at the end of the day, it’s really up to [the imaginary person] to say if she wants anybody included or not.” (P12)*.

Some participants expressed the view that families might not appreciate the extent of the suffering their loved one with mental illness is experiencing, or that they may *“try to sway them”* (F1) to keep them alive, threatening their autonomy, as this participant identified,

*“So, it– my fear would be that if more people were involved, it would no longer feel like the person’s decision.” (F1).*

Another participant similarly commented:

*“So, at that point, obviously, your family members and friends are going to be like, ‘No, I don’t want you to die. This is crazy.’ But when you’re at that point and you’re signing up for it, you know that it’s the right thing and it makes the most sense. So I don’t think they should have any say.” (P14)*.

On the other hand some family members expressed the view that they should be part of the application process, as they may be able to give important input as to the duration of suffering and quality of life:

*“Not to make the decision, but at least to know what’s going on, you know? I think it’s good for families to know and be around, to be part of this thing, and if there’s any help that can be provided… you know?” (F9)*.

Finally, both family members and patient participants commented on the value of informing families of a MAiD MI-SUMC process underway, as this participant put forward:

*“If there are people who are going to be affected by it, that they should not find out by accident somewhere.” (P2)*.

### A holistic person-centred assessment process

Patient and family member participants’ perspectives on the MAiD MI-SUMC assessment process were fully aligned, focusing on assessing MAiD MI-SUMC requests using strengths-based holistic assessments, and as this participant outlined, getting to know the patient’s ‘story’:

*“I think he needs to know— the doctor needs to know the story. Not just who the [imaginary person] is sitting in front of him, but how he became– how his mindset at that moment happened to be, because MAiD — it wasn’t, wasn’t there all the time. But the thought might have been.” (F4)*.

We describe below participant perspectives on assessing a MAiD MI-SUMC request, as well as the underlying mental health condition and its impact, including irremediability. Participant narratives underscored hopes for compassion, appreciation of one’s autonomy, consideration of one’s support environment, and an understanding for the social determinants of health in the assessment process.

#### An attentive and compassionate assessment of the MAiD MI-SUMC request

In evaluating a MAiD MI-SUMC request, participants highlighted the importance of exploring the reasons for the request, voluntariness and consistency of the request, and capacity to consent, by getting to know the person and their story. First, participants described the importance of assessors spending some time to understand the reasons underlying the request, to ensure that:

*“It’s the individual themselves who’s requesting [MAID MI-SUMC].” (P5)*.

As another participant described:

*“Listen to [the imaginary person’s] reasoning, listen to where they are in life and the reasons that brought them to that decision, because we know they’re not going to reach that decision lightly.” (F7)*.

Participants also spoke to the need to assess *“clarity of mind”* (F10) and capacity to make treatment decisions. As this participant explained:

*“If [the imaginary person is] thinking straight, OK, so there’s other– you know, it’s just like, ‘okay, we can evaluate the request and either we approve it or deny it.’” (P15)*.

Some participants described the value of pre-assessing capacity to consent, before further evaluation, to ensure that the individual has capacity to make treatment decisions, with one participant, focused on the primacy of patient autonomy, proposing that *“the only real safeguard should be informed consent.”* (P6).

Participants finally spoke of the need to assess the consistency of the request over time to ensure that it is a decision reached over *“a long period of time”* (P16) and not *“like a knee-jerk reaction to something happening or a symptom popping up…”* (F1) This participant further clarified that consistency does not preclude people having moments of ambivalence, but rather avoids a

*“Yo-yo so intense, back and forth, that you can’t be sure the person really knows and understands what MAiD is.” (F1)*.

#### A comprehensive assessment of the underlying condition, social determinants, and their impacts

Both patient and family member participants emphasized the value of comprehensive, person-centred assessments, inclusive of medical history, life circumstances, presence (or absence) of social and family supports, assessment of acute suicidality, and the primacy of the subjective experience of suffering. Participants outlined the need for a diagnostic assessment, to confirm the diagnosis of a mental illness and establish its chronicity, current symptom burden and their impact. As this participant expressed:

*“Well, I guess obviously, they would have to know my complete history with mental illness throughout my life. And like how, how severe it is and how long it took care for, cumulatively but also, if it’s been a — if my current situation with depression has been lasting a long time.” (P17)*.

As part of the assessment, they highlighted the importance of assessing the applicant’s quality of life, and of exploring strategies to bring meaning and joy:

*”Such that you feel you’ve got a reason to get up every morning, and you’ve got a good reason to be around”. (F11)*

They also spoke of the need to explore the presence (or not) of family and social support, and opportunities to augment these supports. As this participant explained:

*“The person is not just the person in front of them — that they’re a sum of all of the love and support and care around them.” (F8)*.

Several participants spoke to the need to assess suicidality, as this participant outlined:

*“I think there should be some assessment, written assessment… that goes with the application to the doctor,” (P9)*.

And as another participant highlighted, assessing suicidality to inform eligibility:

*“I would say the doctor needs to know that probably, one, that he’s not going to be committing suicide…. So that’s a big thing.” (P16)*.

Several participants also commented that health providers should have and display a deep understanding of the social determinants of health, and their impact on mental health, in their interactions with patients. As this participant outlined:

*“If that doctor can’t empathize to the point of understanding what it’s like to have $150 for the month after you pay the stuff that you need to pay that still isn’t counting any of the stuff you really need, right? And if the doctor, you know, can’t empathize with what it’s like to just be utterly alone and unappreciated, then they are not gonna really understand the pieces that are fixable, the things that could be augmented with a basic income program, with a good peer-support worker or connection group, with like… god knows.” (P11)*.

Finally, participants spoke to the experiences of suffering, which they perceived as feeling *“overwhelmed”* (F9), or experiencing *“constant emotional pain”* (P18) which is unbearable, and is the result of a mental disorder rather than social reasons or stresses. They identified the primacy of the applicant’s own assessment of their suffering, as this participant expressed:

*“‘I perceive my own suffering and my own anguish as intolerable to the point where I wish to end my life.’ That should be the baseline of eligibility.” (F7)*.

#### Assessing irremediability in the context of treatment, social support, and hope

Discussions on irremediability focused on treatment history, treatments and supports yet to try, and ensuring input through expert consultations. Many participants commented on the importance of obtaining a detailed history of the course of illness and its response to treatment, including professional mental health supports, as well as social supports and recreational activities. As this participant highlighted, the assessment should cover:

*“What has [the imaginary person] done to [get] relief from symptoms, what healthcare workers he has worked with for that, when is the last time he had some treatment or change of medication, what kinds of results he has got from the different treatments” (F12)*.

Several participants commented on the impact of the social determinants of health on symptom burden and how addressing those first might be key to determining irremediability:

*“And, I think another thing is, like, if [the imaginary person’s] symptoms, say he was in a situation where he’s — he had stable housing, he had an outreach worker come and see him every week and a family doctor and maybe a support group that he was going to and his symptoms didn’t get appreciably better, like the circumstances didn’t — his improved circumstances didn’t improve his symptoms, that’s something to consider too.” (P2)*.

Some participants linked irremediability with finding hope (or not), with contrasting views expressed regarding hope in the context of mental illness. As this participant described:

*“[The clinician] needs to know beyond simple diagnostic criteria, into ‘is there any reason that this person wouldn’t be presented with hope?’ He needs to be — he/she, the clinician, needs to be honest with themselves about ‘what is the actual likelihood that I can put him on any path to effect any changes?’” (P11)*.

Other participants contended that in mental health there is always hope:

*“It can’t possibly be based on black-and-white objective reality of there being no hope, because that isn’t true. It just isn’t true, right? It’s, and for mental health, it isn’t hopeless”. (P13)*

Yet other participants linked remediability and consequently hope to specific mental health conditions:

*“The doctor should know that depression and posttraumatic stress are not incurable conditions.” (F13)*.

Lastly, participants commented on treatments not yet attempted and new and emerging treatments that may provide relief. They described that assessors would ideally inform MAiD MI-SUMC applicants of experimental treatments and refer them to potentially helpful treatments as well as peer support. As these participants expressed:

*“[…] you know — is there anything else that we could offer [to the imaginary person]? A physician should be offering absolutely everything” (F14)*.

And:

*“They can make referrals to services that will help [the imaginary person]” (P6)*.

And:

*“What kind of supports can you put in place to sustain this? Are there available DBT classes that [the imaginary person] can… participate in, so that when she does have these triggers, she has these tools and these individuals to help? Peer support. Peer support is huge.” (F5)*.

### Need for support in the aftermath of the decision

Patient and family member participants were aligned in their views of key considerations in the aftermath of a MAiD MI-SUMC assessment, including the value of an appeal process. We present below participant perspectives on main steps to be implemented following an approved or denied MAiD MI-SUMC request.

#### An informed and supportive process after request approval

Patient and family member participants commented on the information and support needs of approved MAiD MI-SUMC applicants, their need for ongoing care, provisions for possible request withdrawals, and support for families.

Recognizing the emotional and social impact of an approved request, including the need to make final arrangements and say good-bye to friends and family, participants expressed that assessors would need to offer detailed information about next steps following the MAiD MI-SUMC eligibility assessment, while putting patient preferences first, and minimizing suffering, as these participants expressed:

*“All on your terms, ‘what you want to do?’, ‘how you want to do it?’” (P19)*.

And:

*“You know, you don’t want to create more suffering or harm in the process of offering this, so just making sure that [the imaginary person is] in a state where whatever path they’re choosing for MAID won’t create more suffering in the end.” (F1)*.

Reflecting on the mandatory 90-day period between the onset of eligibility assessment and the receipt of MAiD, the majority of participants supported a waiting period following a positive eligibility assessment outcome that varied in length to up to a year or more, because *“it’s a big decision”* (P15). Others suggested no wait time after completion of the eligibility assessment, or a wait time chosen by the patient. They discussed the importance of ongoing treatment and supports throughout the process, with periodic check-ins, re-assessments of capacity, and reminders of the voluntariness of the request and hence the possibility of a MAiD MI-SUMC request withdrawal.

Although some envisioned that efforts during this period might be geared towards changing applicants’ minds and helping with MAiD MI-SUMC request withdrawals, others described individual or group supports and interventions to set applicants’ affairs in order, or in preparation for death and dying, as this participant explained:

*“I think there should be continuous support maybe with a social worker, or a support group, or something of that, where [the imaginary person] can go to and just talk about being approved, maybe the challenges, or surprising things, or just the weird things that she is feeling now that she comes closer to the end of her life.” (F15).*

Participants also commented that request withdrawals should be treated with compassion and respect, with a wide range of opinions among both patient and family member participants regarding implications of such withdrawals for future eligibility. Although some proposed that a request reversal should just put things on hold, with the ability to resume the MAiD MI-SUMC process where it was left off at any time, others commented that a withdrawal should impact future eligibility to reapply, or necessitate a waiting period before re-applying. There was consensus that following a request withdrawal, applicants should be connected to services and supports to understand the rationale for their decision and to develop appropriate care plans, including access to new or untried treatments.

Finally, participants commented on the need for instrumental supports around estate planning, funeral arrangements, arranging for organ, tissue or body donations, and informing family and designated loved ones. Family member participants additionally identified the need for family supports, as this participant described:

*“As a parent, grief and processing of grief and the anticipation of loss. And, I think, support for the family in all instances of MAiD, I think it’s important.” (F8)*.

#### Extra support and alternate options after request denial

Participants described the need for clearly communicating the rationale for denying a MAiD MI-SUMC request, focusing on hope rather than failure to meet eligibility criteria. As this participant outlined:

*“I think that, first, it should be very clearly explained why the request was denied to [the imaginary person] and ensure that she has a good understanding of that as well, and is able to appreciate the different points that led to the decision. She should be offered some of the other treatments that are available, not coercively, but just ensure that she is aware of them and link to a health care professional or– who could provide that.” (P4)*.

Several participants additionally identified the need for closer ties to friends and family, as well as referrals and connections to recreational activities, social support, peer support, income support and housing as essential to improving applicants’ quality of life. As these participants said:

*“Some kind of peer support, some kind of connection to community” (P11)*

*“And trying to get [the imaginary person]’s family more involved, and his friends more involved.” (F16)*.

Both patients and family member participants described an expectation of marked distress, including increased suicidality, and either increased efforts to get well or attempts to worsen one’s situation so they become eligible for MAiD MI-SUMC following a denied request. In this context, there were suggestions for follow-ups and wellness checks to monitor for suicidality, with a range of views expressed on managing suicidality. Some participants identified ongoing suicidality as a reason to reassess and grant a MAiD MI-SUMC request, as a preferred way to die, as this participant expressed:

*“Maybe the compassionate thing to do is to grant the medically assisted death, [the healthcare providers] could do that — and then change their opinion and grant it.” (P9)*.

Some participants suggested that treating providers should be held accountable, or even “lose their license” (F13), if a person that has been under their care requests MAiD MI-SUMC and commits suicide after being denied. Other opinions proposed that MAiD assessors should also anticipate such outcomes and bear accountability:

*“So, I definitely think there needs to be some accountability around denying [the imaginary person] and not looking into the more complex factors of what happens if he does get denied.” (P6)*.

Others suggested that acute suicidality following a denied request should trigger existing suicide prevention strategies, including involuntary hospitalization to keep someone safe, as this participant described:

*“After being denied, I would hope that there would be a mandatory stay as an inpatient and be monitored by professional mental health staff to make sure that when she’s released she’s stable and rational, of sound mind, and not emotional.” (P18)*.

Some participants proposed an appeal process and access to alternate opinions, following a denied request. The majority of participants favored the right to reapply for MAiD MI-SUMC, with proposed wait periods ranging from weeks to several years. Finally, a few participants questioned the legal framework and the basis upon which providers could find a person ineligible, as this participant exemplified:

*“Why would they have the right to deny? What would they use as a basis? Like, is there a checklist? And if there is a checklist, who’s behind it? What person, what medical professional, has enough background and everything that could possibly make a person want to do this or come to the conclusion that they don’t qualify?” (F4)*.

## Discussion

The views and values of patients and families are often overlooked in service planning, leading to programs and services that do not address their needs and preferences [39, 40]. This study, using rigorous qualitative methods and participatory engagement processes, adds to the limited literature on the perspectives of persons with mental illness and their families on MAiD MI-SUMC care processes, and their implications for practice. Persons with mental illness and family participants were aligned in their views on MAiD MI-SUMC service delivery, with a variety of perspectives expressed within both groups in our study [35, 41, 42]. Participants called for MAiD public awareness raising and patient psychoeducation, assessment teams and processes that unpack “the story” and minimize suffering, with special attention to the assessment aftermath. They also identified key care ingredients throughout the MAiD MI-SUMC process, including a person-centred non-judgmental stance by care providers, inter-professional holistic care, shared decision-making, and the primacy of patient autonomy. We discuss below MAiD MI-SUMC practice implications.

First, regarding public awareness and psychoeducation, participant narratives aligned with prior research suggesting that patients value clinician openness in discussions on MAiD, transparency about the legal framework and the assessment process, and maintenance of clinician-patient relationship, even when clinician and patient disagree about MAiD [43]. Although there was widespread agreement on the value of clinicians responding to patient-initiated MAiD MI-SUMC questions using a non-judgmental stance, participants voiced conflicting views on clinicians introducing MAiD in goals of care discussions with persons with a mental illness. In balancing potential harms with the right to self-determination, the Canadian Association of MAiD Assessors and Providers supports informing eligible patients about MAiD, highlighting the value of informed consent, the importance of patient autonomy, and the role of clinicians as gatekeepers of clinical information [44]. Given participant concerns about clinician-initiated MAiD MI-SUMC conversations, care providers will need clear guidance on how best to promote unbiased MAiD MI-SUMC information sharing and person-centred, respectful and inclusive goals of care discussions. Care providers will also need to consider the implications of approving or providing an intervention that they are restricted in freely proposing. Practice guidelines may be helpful in addressing this clinical paradox and in promoting consistent approaches across care providers [45, 46], while training of peer support workers, identified by participants as important contributors to MAiD discussions, may also be valuable.

Secondly, participants cautioned that the MAiD MI-SUMC assessment process may exacerbate suffering, through interactions with providers who may be invalidating, assessment processes that may undermine autonomy, or through an unfavorable request outcome, a notion supported by prior research [47, 48]. They underscored the importance of hope in alleviating suffering and suggested ongoing and additional services and supports to MAiD MI-SUMC applicants throughout the assessment period. This perspective aligns with literature suggesting that a MAiD request may be at first considered as “a cry for help”, and with a two-track approach focusing both “on the life track” by engaging in ongoing treatment, and on the “death track” by assessing MAiD eligibility [22, 23, 44]. Similarly, in keeping with prior research with patients with other health conditions and their families, participants stressed the importance of holistic, interdisciplinary assessments, and the opportunity to tell their story and exercise autonomous health decision making [25, 49]. These perspectives support and are supported by prior research calling for MAiD care coordination, inter-professional approaches, and attention to applicants’ and families’ emotional, social and spiritual needs, among others [24, 25, 50–52]. They also align with published protocols leveraging additional safeguards in the assessment process, such as comprehensive evaluation by multiple psychiatrists, and a prospective multi-expert panel to review MAiD requests [22, 23].

The majority of participants did not identify specific diagnostic categories as more likely to be eligible for MAiD MI-SUMC, but rather viewed the grievousness of a condition and the burden of suffering, in all its dimensions [47, 53, 54], as subjective experiences. Although they acknowledged the need for experts to assist in assessing irremediability, ultimately, they focused on the patient’s lived expertise, attempted treatments, the role of social determinants and their interaction with illness experience and trajectories, and shared decision-making in MAiD MI-SUMC eligibility assessments. Participants’ perspectives on illness trajectories align with recent work on the philosophy of psychiatry on externalism of mental disorders, emphasizing the relationship between an individual and their external environment in the precipitation and perpetuation of mental illness and, hence, irremediability [55, 56]. Further, these perspectives are in contrast to irremediability being related, in part, to the course of the specific illness, independent of the suffering to date, which is difficult to predict on an individual basis.

Of note, the majority of individuals who received physician assisted death in the Netherlands were diagnosed with depression, personality disorder, or trauma and stress-related disorders [57].

Thirdly, participants identified support needs for both patients and families during and following the MAiD MI-SUMC assessment, including, in alignment with prior studies on MAiD for conditions other than mental illness [26, 58, 59], instrumental supports with end-of-life planning, and bereavement supports for families [60]. In the case of denied applications, they also identified the need for clinical supports for MAiD MI-SUMC applicants, including monitoring for suicidal ideation. The literature suggests that many MAiD applicants report ongoing suicidal ideation following a denied MAiD request [61], calling for follow-up assessments and safety planning following the MAiD assessment. Notably, several study participants viewed suicidal ideation as a reason to grant, rather than refuse MAiD MI-SUMC, considering it a better, more dignified option to the painful and uncertain death by suicide for those intent on ending their life [62, 63]. These participants placed greater emphasis on patient choice and self-determination that is articulated in MAiD legislation in Canada, with views and perspectives in keeping with de-medicalized assisted suicide services, where access to assisted death is patient determined [64]. However, other approaches to care and support might be possible. When ongoing interventions are not effective, create more harm than benefit, and patients report intolerable suffering and poor quality of life, re-orientation of their goals of care toward a palliative approach can be considered [65]. Accordingly, the implications of our findings resonate with recent scholarship on ‘palliative psychiatry’, which focuses on palliative goals of care, such as improving quality of life and relief of suffering, for patients with serious mental illness who are not necessarily at the end of life [66, 67]. Taken together, this calls for setting balanced expectations for patients and families through public awareness campaigns and goal of care discussions, along with transparent communication that MAiD MI-SUMC assessments include the consideration of patient needs and preferences in tandem with legal and medical criteria and safeguards.

This study has many strengths, including rigorous qualitative methods, and participatory engagement with experts with lived experience. The study nonetheless has some limitations, including lack of representation of certain communities, such as Indigenous peoples, LGBTQ2S + participants, immigrants, as well as representation from people with diverse illness trajectories, despite efforts to promote diversity in our sample. Finally, the study took place in a country where MAiD is accepted and legally available for chronic health conditions other than mental illness, and where MAiD MI-SUMC is anticipated to be available in March 2024, thus responses are framed within that presumption. Nevertheless, the study amplifies patient and family perspectives on an intervention that remains controversial in the field of psychiatry and adds to the limited research in this important area. Future work should further examine key ingredients of effective patient-clinician communication in discussing MAiD MI-SUMC, unpack the concept of MAiD as a harm-reduction strategy, and pursue rigorous evaluation of MAiD MI-SUMC in jurisdictions where it is legally permissible, including cross-cultural comparisons, in partnership with patients and family members.

## Conclusion

Research on patient and family perspectives on MAiD MI-SUMC implementation and care processes can complement existing and emerging professional practice standards and help inform care delivery. Our findings highlight the primacy of person-centred holistic assessment paradigms, and the importance of shared decision making and patient autonomy in MAiD MI-SUMC considerations. Our findings also identify the need for ongoing public education, the development of practice guidelines to bring together the diverse perspectives of all stakeholders, and assessment processes that minimize suffering and attend to the eligibility assessment aftermath. Patient and family perspectives will continue to be essential in MAiD MI-SUMC implementation efforts, to better address the needs of diverse communities and inform MAID MI-SUMC evaluation and improvement efforts.

### Electronic supplementary material

Below is the link to the electronic supplementary material.

Supplementary Material 1

## Data Availability

The datasets used and/or analysed during the current study available from the corresponding author on reasonable request with the approval of the governing Research Ethics Board.

## Abbreviations

MAiD: Medical Assistance in Dying
MAiD MI: SUMC–Medical Assistance in Dying where a Mental Illness is the Sole Underlying Medical Condition
