# Supplementary material for: Care considerations in medical assistance in dying for persons with mental illness as the sole underlying medical condition: a qualitative study of patient and family perspectives
Source: BMC Psychiatry. 2024 Feb 14;24:120. doi: 10.1186/s12888-024-05541-5 (PMC10865571; doi:10.1186/s12888-024-05541-5)
Supplement: Supplementary file 1 — Supplementary Material 1 [file 12888_2024_5541_MOESM1_ESM.docx]

**Table S1: MAiD Eligibility Criteria and Safeguards for Persons whose Natural Death is not Reasonably Foreseeable^1^.**

As of March 17, 2021, persons who wish to receive MAID must satisfy the following eligibility criteria:

- be 18 years of age or older and have decision-making capacity
- be eligible for publicly funded health care services
- make a voluntary request that is not the result of external pressure
- give informed consent to receive MAID, meaning that the person has consented to receiving MAID after they have received all information needed to make this decision
- have a serious and incurable illness, disease or disability (excluding a mental illness until March 17, 2024)
- be in an advanced state of irreversible decline in capability
- have enduring and intolerable physical or psychological suffering that cannot be alleviated under conditions the person considers acceptable

**Safeguards for persons whose natural death is not reasonably foreseeable**

The following procedural safeguards apply to persons’ whose natural death **is not** reasonably foreseeable (*indicates safeguards specific to those requests):

- request for MAID must be made in writing: a written request must be signed by one independent witness, and it must be made after the person is informed that they have a “grievous and irremediable medical condition” (a paid professional personal or health care worker can be an independent witness)
- two independent doctors or nurse practitioners must provide an assessment and confirm that all of the eligibility requirements are met
  - *if neither of the two practitioners who assesses eligibility has expertise in the medical condition that is causing the person’s suffering, they must consult with a practitioner who has such expertise
- the person must be informed that they can withdraw their request at any time, in any manner
- *the person must be informed of available and appropriate means to relieve their suffering, including counselling services, mental health and disability support services, community services, and palliative care, and must be offered consultations with professionals who provide those services
- *the person and the practitioners must have discussed reasonable and available means to relieve the person’s suffering, and agree that the person has seriously considered those means
- *the eligibility assessments must take at least 90 days, but this period can be shortened if the person is about to lose the capacity to make health care decisions, as long as both assessments have been completed
- immediately before MAID is provided, the practitioner must give the person an opportunity to withdraw their request and ensure that they give express consent

^1^https://www.justice.gc.ca/eng/cj-jp/ad-am/bk-di.html#s1-2
